# Case of Fracture of the Left Parietal Bone, from the Expulsive Action of the Uterus

**Published:** 1846-01

**Authors:** J. D. Wharrie

**Affiliations:** Hamilton


*Case of Fracture of the Left Parietal Bone, from the Expulsive Action of the Uterus.* By J. D. WHARRIE, M. D., Hamilton.—The body of a child having been found secretly buried near the Calder, it was reported to the Fiscal, when Mr. Jamison, Surgeon at Bellshill, and I, were appointed by the Sheriff to make a *post-mortem* inspection of the body. There was handed over to us a coarse deal box, nailed up, on opening which we found the body of a female child, wrapped up in some clothes. Before proceeding to dissection, we carefully examined it, and observed no marks of injuries, except that the cranium near the posterior fontanelle had a puffy feel, as if from containing extravasated blood, and when pressure was made on the forehead, blood issued from the right nostril. The muscular substance of the body was firm, but the cuticle in parts easily peeled off, as if from incipient putrefaction; from the weather being cold, we conjectured two or three weeks had elapsed since delivery. The naval cord was cut and tied, six inches of it still remaining attached to the body. The child weighed seven pounds, and measured in length twenty-one inches. The chest had a flat appearance, and there were no marks around the anus of meconium. On opening the body, we found the lungs of a dark colour, with their edges sharp, occupying but a small space in the posterior part of the chest, and not covering the heart or pericardium; and the diaphragm was arched upwards. The lungs, with the heart, being removed from the body, and placed in water, sank to the bottom of the vessel; both lungs, previously cut into pieces, were then subjected to the same trial, and all of them immediately sank; none of these portions had the slightest crepitous feel, nor did they emit air bubbles, when pressed; the pulmonary vessels appeared also to contain very little blood. There was no blood in the right side of the heart, but a small quantity was found in the left side; and the foramen ovale was open. We examined the mouth, windpipe and gullet, and found all of them in a natural state. On proceeding to dissect off the integuments from the scalp, we found on the left side of the head about two teaspoonfuls of blood extravasated under the pericranium, at (that part of the head which felt puffy previous to dissection) there was also a little blood, though smaller in quantity, effused immediately adjoining this on the right side of the head. On removing the blood on the left side, we discovered a fissure fully half an inch in length, in the edge of the left parietal bone, close to the line of the sagittal suture, and near the posterior fontanelle. There was not the slightest depression of the bones at the seat of the injury; nor, on minutely examining the scalp, by shaving off the hair, could we detect any discoloration, or the slightest mark indicating a blow The brain was rather in a soft state; there was no extravasation of any consequence upon its surface, or within its substance, but the extremities of some of the blood vessels seemed to have given way to a trifling extent. From the size and general appearance of the child, there could be no doubt, that it was born at the full period of pregnancy, and it was also quite evident that it had not respired, not even feebly. The important question comes to be—What was the immediate cause of death? I suspect—simply the violent expulsive efforts of the uterus, forcing the child forward, and producing severe pressure on the cranium; or it is possible, a portion of the naval cord might have fallen down, and been compressed. At all events, there were no marks by which the woman could be accused of infanticide, nor, as afterwards appeared, of concealment of pregnancy.

The mother in her declaration said,—" That she was 28 years of age, was never married, but had borne three children, her last only was still-born, and that its birth took place about a month previous to her examination;—that she was in labour from Saturday afternoon till Tuesday morning about 11 o'clock; she was very ill from Monday night, but until that time she was only in a lingering way, and there was no person attending her during all the time except her mother, as she had no money to pay for a doctor or midwife;—that she knew her mother could do any thing that was required, as she had been with women before, and with herself when she had her other two children, assisted by neighbours;—her mother was the only person present at the time when the child was born; that it was laid in the bed, till the old woman hurried out for two neighbours;"—These neighbours declared they never saw the child more. She farther said, that they kept the dead body till Thursday morning, having no money to pay the expense of burying it;—her brother made a box, into which it was put, and her mother carried off the box, saying, "she would bury it at the side of the Calder, till she got 'baubees,' to bury it regularly;" but in the mean time it was accidentally discovered.

On the whole affair being properly investigated, and the precognition taken sent to the Crown Counsel, the opinion given was, that there ought to be no farther proceedings.—*Lond. and Ed. Monthly Journ.*

